# Metagenomic Approaches for Understanding New Concepts in Microbial Science

**DOI:** 10.1155/2018/2312987

**Published:** 2018-08-23

**Authors:** Luana de Fátima Alves, Cauã Antunes Westmann, Gabriel Lencioni Lovate, Guilherme Marcelino Viana de Siqueira, Tiago Cabral Borelli, María-Eugenia Guazzaroni

**Affiliations:** ^1^Department of Biochemistry, Faculdade de Medicina de Ribeirão Preto, University of São Paulo, Ribeirão Preto, SP, Brazil; ^2^Department of Cell Biology, Faculdade de Medicina de Ribeirão Preto, University of São Paulo, Ribeirão Preto, SP, Brazil; ^3^Department of Biology, Faculdade de Filosofia, Ciências e Letras de Ribeirão Preto, University of São Paulo, Ribeirão Preto, SP, Brazil

## Abstract

Over the past thirty years, since the dawn of metagenomic studies, a completely new (micro) universe was revealed, with the potential to have profound impacts on many aspects of the society. Remarkably, the study of human microbiome provided a new perspective on a myriad of human traits previously regarded as solely (epi-) genetically encoded, such as disease susceptibility, immunological response, and social and nutritional behaviors. In this context, metagenomics has established a powerful framework for understanding the intricate connections between human societies and microbial communities, ultimately allowing for the optimization of both human health and productivity. Thus, we have shifted from the old concept of microbes as harmful organisms to a broader panorama, in which the signal of the relationship between humans and microbes is flexible and directly dependent on our own decisions and practices. In parallel, metagenomics has also been playing a major role in the prospection of “hidden” genetic features and the development of biotechnological applications, through the discovery of novel genes, enzymes, pathways, and bioactive molecules with completely new or improved biochemical functions. Therefore, this review highlights the major milestones over the last three decades of metagenomics, providing insights into both its potentialities and current challenges.

## 1. Introduction

**Figure 1:**
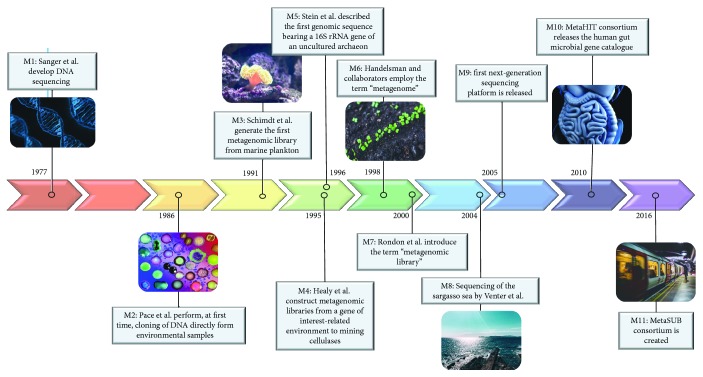
Timeline of the major advancements in metagenomics. Timeline highlighting important developments in the field over the last 40 years, since Sanger sequencing (M1), and over the last 30 years, since the first published metagenomic experiment (M2). The main metagenomic milestones are shown as M1–M11 (all of them are highlighted in the text where they were mentioned).

About thirty years ago, in 1986, Pace and collaborators [1] proposed, for the first time, the revolutionary idea of cloning DNA directly from environmental samples to analyze the complexity of natural microbial populations (Figure 1, indicated as M2). The adopted strategy was based on shotgun cloning of 16S rRNA genes using purified DNA from natural samples. At that time, authors stressed that although the DNA was originated from a mixed population of microorganisms, the methodology allowed the recovery and subsequent sequencing of individual rRNA genes. Thus, by evaluating complete or partial rRNA sequences, the composition of the original microbial populations could be retrieved.

Around ten years later, in 1998, the term “metagenome” appeared, when Handelsman and collaborators [2] described the importance of soil microorganisms as sources for new natural compounds (Figure 1, indicated as M6). According to them, a new frontier in science was emerging—the mining for novel chemical compounds from uncultured microorganisms, which comprises more than 99% of the microbial diversity [3]. This new concept in microbial science opened the mind of the scientific community with respect to the astonishingly large catalogue of biochemical functions available in nature remaining to be discovered.

**Figure 2:**
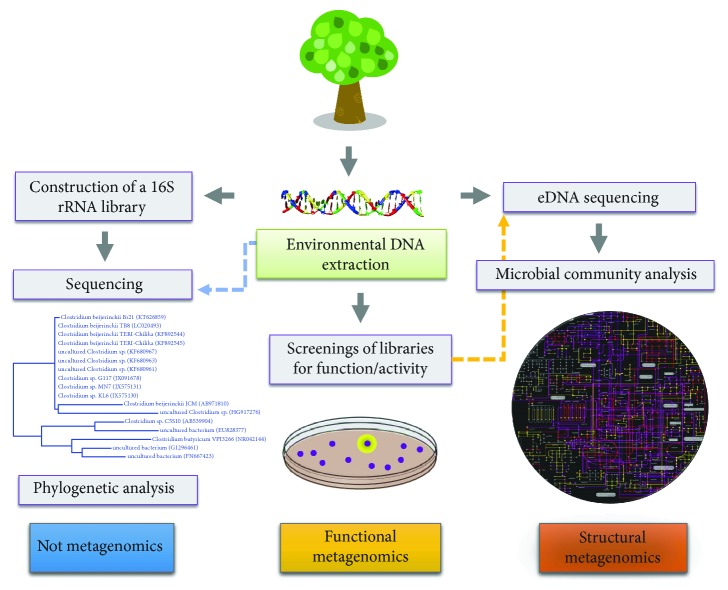
The metagenomics framework and its two main approaches. Both structural and functional metagenomic approaches are the main strategies for exploring key ecological and biotechnological features in environmental samples, respectively. Additionally, 16S rRNA gene surveys can work in synergy with metagenomics for further understanding of microbial ecology.

Currently, metagenomics is subdivided into two major approaches, which target different aspects of the local microbial community associated with a determined environment. In the first one, the so-called structural metagenomic approach, the main focus is to study the structure of the uncultivated microbial population, which can be expanded to other properties, such as the reconstruction of the complex metabolic network established between community members (Figure 2) [4, 5]. In this sense, the microbial community structure can be defined as the population composition and its dynamics in a specific ecosystem, in response to selective pressures and spatiotemporal parameters. The study of the community structure allows a deeper understanding about the relationships between the individual components that build a community and is essential for deciphering ecological or biological functions among its members [5, 6]. In a different manner, the functional metagenomic approach aims to identify genes that code for a function of interest, which involves the generation of expression libraries with thousands of metagenomic clones followed by activity-based screenings (Figure 2) [7, 8].

It is important to highlight that 16S rRNA gene surveys are often referred to as metagenomic studies, although they are not. In the 16S rRNA gene analysis, the study is focused on a single gene used as a taxonomic marker (Figure 2). On the other hand, structural metagenomics aims to investigate the genomes of the microbial community members. In this sense, the later approach allows the overall reconstruction of the community structure, potentially revealing metabolic pathways of the whole microbiome and assigning minor or major geoecological roles to community members [4, 6, 9, 10]. We highlight that 16S rRNA surveys and metagenomics are not mutually exclusive; on the contrary, approaches that establish a link between 16S rRNA analyses to genes or metabolic pathways have been shown to be useful in determining the functional potential of a microbiome [11–13]. Thus, the combination of these complementary strategies allows for a deeper exploration of relevant biological questions in microbial ecology such as “who are the members of the community?” and “what are their functional roles?”

At the beginning of the metagenomic studies, the use of Sanger sequencing technology [14] provided important progresses in the field [15–17] (Figure 1, indicated as M1). However, the advent of next-generation sequencing (NGS) technologies capable of sequencing millions of DNA fragments simultaneously, at a low cost, greatly bolstered the field [18–21] (Figure 1, indicated as M9). Comparatively, NGS platforms can recover up to 5000 Mb of DNA sequence per day with costs at about 0.50$/Mb, while Sanger sequencing methodology allows about 6 Mb of DNA sequence to be created per day with costs at about 1000 higher [22].

This review focuses on how metagenomics has contributed to gain scientific comprehension in many different areas of knowledge. In this manner, milestones in metagenomics have ranged from findings with significant biotechnological impact to unexpected outcomes with high biomedical relevance, shining a light on hidden molecular components and on the connections between microbial communities and complex diseases [23–26]. We also discuss the current boundaries of the field that should be overcome for the achievement of conceptual advances in microbial science.

## 2. Milestones in Metagenomics

In 1991, Schmidt and collaborators generated the first metagenomic library using DNA from marine picoplankton [27], and, some years later, Healy et al. constructed metagenomic libraries from an enriched consortia sampled from cellulose digesters to mine genes encoding cellulases [28] (Figure 1, indicated as M3 and M4, resp.). In this context, the idea of screening metagenomic libraries from specific environments was introduced, allowing that the number and properties of the retrieved genes (e.g., enzymes) could be correlated to the conditions of the source environments. However, only in 2000, Rondon and collaborators [29] coined the term “metagenomic libraries,” by generating libraries in BACs (bacterial artificial chromosome) using DNA from soil samples (Figure 1, indicated as M7). Furthermore, the authors also performed phylogenetic analysis of 16S rRNA sequences and identified a number of clones expressing heterologous genes in functional screenings using *Escherichia coli* as host.

Since then, a large amount of data has been generated using metagenomic approaches and impacting different areas of high applicability in our society (Figure 2). Here, we highlighted the main milestones in metagenomic studies that defined the field in distinct contexts. Although many of the advances in the field can be credited to novel sequencing approaches and the development of new computational methodologies to analyze the generated data, in this review, we have focused on highlighting biological discoveries through metagenomics rather than describing these more technical strategies, which have been addressed in recent reviews [30–32].

### 2.1. Genomic and Taxonomic Novelties in Environments

Ever since the proposal of using molecular markers such as 16S (or 18S) rRNA for comparing species similarity, in the late 1970s, followed by the outlining of a third “urkingdom” (the Archaebacteria), our knowledge of organism relatedness has taken a great leap forward. Taxonomic classification started to rely on a “comparative approach that can measure the degree of difference in comparable structures,” which not only allowed a more resolved phylogeny and a less biased organization based on the *Prokaryotae* versus *Eukaryotae* dichotomy but also made it possible to better understand how life on earth has come to be [33]. In this context, metagenomic approaches have been used to generate data of novel genomes from otherwise uncultivated organisms, deepening the framework of genomic tools available for comparison and study.

The shotgun sequencing of the Sargasso Sea waters, by Venter and colleagues in 2004 [34], is one of the most illustrative examples of how metagenomics are a feasible way to accumulate genomic knowledge (Figure 1, indicated as M8). In this study, Venter and collaborators have recovered almost 1.5 Gbp of microbial DNA sequences from microbial populations of three marine sites using en masse whole genome shotgun sequencing from filtered sea water. This leads to the finding of almost 70 thousand novel genes among the roughly 1.2 million genes by ORF (open reading frame) identification and alignment of the putative protein products. Among the main findings, the researchers described a novel ammonia oxidation pathway uninhibited by UV light, putative genes for trace metal resistance such as arsenate and copper, and, additionally, up to 782 new proteorhodopsin-like receptors genes. The latter added an insight to the exotic marine photoheterotrophic lifestyle first described by Béjà and colleagues only a few years before [35]. Further, the metagenomic study of the Sargasso Sea allowed the identification of 148 previously unknown rRNA bacterial phylotypes. However, this probably was an underestimation of the environment's total genomic pool, given that the difference in the number of rRNA coding genes within the rRNA operon between species may result in biases in PCR studies with under representation of some of the community constituents, as also discussed by Klappenbach and colleagues [36].

In this context, a recent study based on metagenomic data has shown that about 10% of environmental bacterial or archaeal sequences might not be recovered when using a targeted PCR survey with the most common primers for SSU rRNA [37]. For instance, a study led by Brown and colleagues in 2015 [38] first described the Candidate Phyla Radiation (CPR) bacterial lineage comprising at least 25 new bacterial phyla, which make up to at least 15% to the Bacteria domain. These novel organisms lack many biosynthetic pathways and possess unusual features in their small genomes, such as self-splicing introns within the rRNA genes and novel ribosome structure, providing insight to the organelle's evolution trajectory. It is worth noting that all complete CPR genomes curated in the work have only one copy of the 16S rRNA gene and many of the organisms would evade detection by 16S rRNA gene amplicon surveys, even though corresponding to such a high percentage of bacterial diversity in environments. In their work, Venter and collaborators have attempted to address this issue and to better elucidate the phylogenetic relationships in the recovered genetic material by employing six other phylogenetic markers, such as heat shock protein 70 (HSP70) and elongation factor Tu (EF-Tu), as well as several methods to estimate species diversity, which have resulted in the estimation of over 1000 species [34].

Metagenomic studies also shed a light on challenges in the field of evolutionary biology, such as in the understanding of sexual reproduction as a constraint on genomic variation [39]. Previous to the metagenomic era, it was believed that many microbial species should be genomic clones—asexual reproduction was assumed to produce identical clones. Yet, metagenomic analyses unexpectedly revealed that most microbial species were not clonal [34]. Thus, asexual reproduction present in bacteria should increase genetic variation providing evolutionary diversity for future environmental challenges [40]. This finding was essential to support the conclusion that sexual reproduction acts as a constraint on genomic and epigenetic variation, thereby limiting adaptive evolution [39].

In recent years, new sampling and sequencing techniques allowed researchers to further explore life diversity improving phylogenetic, genomic, and ecological notions previously established. Rinke and colleagues, for instance, revealed the “microbial dark matter” in a single-cell genomics study that comprised over 20 major uncultivated archaeal and bacterial lineages [41]. They first described archaeal sigma factors and the first reported lateral gene transfer from a eukaryote to an archaeon. Another insight of how the archaeal/eukaryotic relationship came to be and how the modern eukaryotic cell was formed was found by Spang and collaborators [42], who have identified a candidate archaeal phylum which forms a monophyletic group with eukaryotes. It also possesses genes encoding proteins similar to those related to cell shape formation processes in eukaryotic cells, which might suggest sophisticated membrane remodeling and vesicular trafficking processes in eukaryotic cells even before the acquisition of mitochondrion.

### 2.2. Innovative Functions in Uncultivable Microbes

Sequenced-based analysis in metagenomics can be accomplished, overall, by following one of the following paths: (i) sequencing all clones with a phylogenetic marker indicating the potential taxonomic source of the DNA fragment or (ii) sequencing random fragments until a gene of interest is found followed by sequencing of the adjacent regions to find taxonomic markers. The former method was developed by Stein's group and described the first genomic sequence bearing a 16S rRNA gene of an uncultured archaeon (Figure 1, indicated as M5) [43]. This provided the first highlights of the metagenomics capabilities for unravelling novel genes, functions, and taxonomic groups. In this study, with a marine picoplankton assemblage collected at eastern North Pacific, a 38.5 kbp fragment containing an archaeal 16S rRNA gene was isolated for the first time. Among other features, the discovery of an RNA helicase and a glutamate semialdehyde aminotransferase, which were still unknown in archaeal organisms, was reported. The metagenomic library in this study had an average fragment size of 40 kb and was based on fosmid backbones. The screening was very labor intensive, relying on Southern blotting with phylogenetic probes for 16S rRNA gene, followed by sequencing of selected clones through automated Sanger or shotgun methods.

It did not take long for the same group to push the boundaries and further advance the incipient field of metagenomics [35, 44]. From the surface waters of the Californian coast, a marine planktonic bacterial assemblage was collected for metagenomics analysis. Hitherto, bacteriorhodopsins were considered unique features of halophilic archaea; however, the small ribosome subunit gene revealed its source as a gammaproteobacterium (uncultivable bacterial SAR86 lineage). It was also shown that the protein was functional when cloned into *E. coli*, presenting similar kinetics to the archaeal-related cognates [35]. In this study, both metagenomic library and fragment average sizes were much larger than in the previous study and the screening process was accelerated (6240 screened clones with an average size of 80 kb) [44]. These improvements were only possible due to the establishment of BACs and PCR-based screening as new tools for metagenomic studies. Thus, it did not take long for novel proteorhodopsins to be detected in other populations of planktonic marine bacteria [45]. This was considered the first great “metagenomic success,” allowing the adoption of these new techniques among laboratories around the world.

### 2.3. Deciphering and Rebuilding Microbial Communities

By starting with an extremely simple microbial community—an acid mine drainage (AMD) microbial community—evidenced by an initial group-specific fluoresce in situ hybridization (FISH) assay, Tyson and collaborators [46] were able to perform the first assembly of genomes directly from environmental samples. In this study, they have obtained two nearly complete genomes of *Leptospirillum* group II and *Ferroplasma* type II and partially recovered another three genomes. The initial characterization of this microenvironment, a biofilm with rather extreme conditions like very acidic pH (approximately 0.83), revealed the presence of archaea (*Thermoplasmatales*) and bacteria (*Leptospirillum*, *Sulfobacillus*, and *Acidimicrobium*), with the domination of *Leptospirillum* group II. The low diversity of the system was reported as possibly related to the extreme environmental conditions. Afterwards, the DNA sequencing of a biofilm sample suggested adaptive molecular traits of the community to survive in this environment—such as homologous recombination forming mosaic genome types—and metabolic adaptations, such as abundance of genes related to ferrous iron oxidation.

In other metagenomic studies, Tringe et al. [5] performed comparisons of the composition and functionality of microbial communities from two nutrient-poor and two enriched-nutrient environments. This approach was mainly concerned with gene function rather than genome composition, overcoming limitations to genome assembly from complex environments. Authors showed that gene function and structure differ in nutrient-limited (Sargasso Sea and AMD) versus nutrient-abundant (Minnesota farm soil and deep-sea “whale fall” carcasses) environments. Some gene functions were exclusive to specific environments, for instance, (i) cellobiose phosphorylases were only found at the agricultural soil and (ii) light-driven proton pumps are only found at the Sargasso Sea samples, whereas no photoreceptors were found at the deep-sea samples.

The development of functional approaches in large scales also provided novel insights into communities' key metabolic process. A comparative functional profiling of 9 biomes was performed by Dinsdale and collaborators [47], describing how different biological traits play essential roles in each environment. For instance, authors showed an abundance of virulence genes in fish- and terrestrial-animal-associated metagenomes in comparison to the other biomes. In contrast, virome analysis of the total number of biomes showed a more uniform gene functional composition due to phages playing similar roles in different environments [47].

### 2.4. City-Scale Molecular Profile of DNA

The last decades of metagenomics have shed a light on the human microbiome and its profound influence on a wide range of aspects which were previously regarded as solely (epi-) genetically encoded, such as diseases susceptibility, immunological response, and social and nutritional behaviors [48–52]. Although there is still much to learn in this subject, recent studies are targeting not only the inside but also the external part of the human world, also known as microbiomes from human-built environments [53–55]. In this context, a myriad of anthropocentric utensils and physical spaces has been assessed, such as kitchen sponges [56], dollar bills [57], ATMs (Automated Teller Machines) [58], homes [59], hospitals [60], subways [61, 62], food production sites [63], and even the International Space Station [64]. The main goal of this new research branch is to provide a framework for understanding the relationship between human societies and microbial communities, ultimately optimizing both human health and productivity.

A seminal research in the context of urban areas was conducted by Afshinnekoo et al. [61], which sampled different features of New York subways and reported that nearly 1700 microbial taxa were dominated mostly by human skin bacteria and to a lesser extent by microbes from the human gastrointestinal and urogenital tracts. Almost half the DNA present on the subway surfaces matched no known organism. Although results showed that the bacteria found in the subways were mostly harmless, several pathogenic agents, including fragments of the plague and anthrax genomes were detected. This was the beginning of an international consortium called The Metagenomics and Metadesign of Subways and Urban Biomes (MetaSUB) that has been sampling urban microbiomes throughout the world [62] (Figure 1, indicated as M11). An important scientific agenda was also launched in 2017, the microbiomes of the built environment [65]. Its main objective is to assess the current state of knowledge on indoor microbiomes and also to map out research agendas and advise government agencies on how living spaces can be designed “to support occupant health and wellbeing.” Other studies have assessed complementary aspects of this matter, such as the influence of landscape connectivity in microbial diversity [66], the influence of green areas in urban spaces [67, 68], and the consequences of excessive time expenditure indoors in the context of both human health and environmental microbiomes [54, 69].

Altogether, those studies allowed the depiction of a few important conclusions regarding human-built spaces and microbiomes, further explained by Stephens et al. [70]: (i) culture-independent methods are essential for those surveys, (ii) indoor spaces often harbor unique microbial communities, tightly related to the indoor sources—mostly humans and pets, (iii) building design and operation can directly modulate indoor microbial communities, and (iv) it is possible to optimize human health by exposure to certain microbial groups. Consequently, society moved from the old concept of microbes as harmful organisms to a new view in which the interaction between humans and microorganisms can be flexible and directly dependent on our own decisions and practices. Further studies shall reveal novel rules on “good living standards” for both humans and microbes in built environments.

### 2.5. The Human Microbiome

The concept of the human microbiome, embracing the idea that human beings are highly susceptible to the microbial communities that live in and on our bodies, was an indubitable milestone in metagenomics with large repercussion in many areas. In this sense, scientific contributions involving metagenomic approaches rapidly highlighted the evidences showing that these microbiomes play key roles in human health and disease.

The human gut microbiota—the collection of microorganisms that compose the human gut—is composed by up to 10^14^ microorganisms [71, 72] including bacteria, viruses, fungi, and protozoa. Deciphering the function and composition of our microbiome—the collective genomes of the microbial community resident—is a challenge that has been explored by researchers in a series of initiatives like the Human Microbiome Project (HMP), the Integrative Human Microbiome Project (iHMP), and the MetaHIT (METAgenomics of the Human Intestinal Tract) [73–75] (Figure 1, indicated as M10). The findings of these projects have provided valuable data about the function of the human microbiome in different tracts (e.g., nasal, oral, skin, gastrointestinal, and urogenital). Particularly, advances in molecular biology procedures, next-generation DNA sequencing, and *omics* techniques have allowed to access not only to the microbial genetic diversity but also to the understanding of the physiology and the lifestyle of our microbiome. In this sense, it was demonstrated that gut microorganisms perform several elemental functions like synthesis of essential amino acids and vitamins and processing of cellulosic material [76], playing an important role in a number of human health aspects [77].

In a series of studies coming out of the Gordan lab at Washington University School of Medicine in St. Louis, Ley and collaborators [78] showed that obesity has a microbial component. To explore the relation between gut microbial ecology and body fat in humans, authors studied 12 obese people, who were randomly assigned to two types of low-calorie diet. The composition of their gut microbiota was monitored over the course of one year by sequencing 16S ribosomal RNA genes from stool samples [78]. Obtained data showed that two groups of beneficial bacteria were dominant in the human gut, the Bacteroidetes and the Firmicutes. In addition, they showed that the percentage of Bacteroidetes correlated with the percentage of loss weight. In other studies [79], they found that the transplantation of the microbiota from obese mice to lean mice could lead to an increase of body fat in transplanted mice when compared with transplantation from lean mice microbiota.

The other important outcome from the human gut microbiome studies was regarding antibiotic resistance. In order to determine an “antibiotic resistance potential,” Forslund et al. [80] performed a quantitative gut metagenomic analysis of known resistance genes from people of three countries. In this study, authors showed that antibiotic resistance gene abundance in the general human population is correlated with the length of antibiotic usage in livestock [80–82]. In another study, Raymond and coworkers [83] showed that the initial gut microbiome affects its recomposition after antibiotics treatment. They administrated two second-generation antibiotics (cephalosporin and cefprozil) in healthy individuals and showed that antibiotics altered the microbiome of healthy volunteers in an interindividual manner, allowing the emergence of potentially pathogenic *Enterobacteriaceae*—in a subgroup of patients—probably related to a decreased initial gut microbiome diversity in those individuals.

Besides obesity and antibiotic resistance, the human gut microbiome has been associated with several diseases as type 2 diabetes, cardiovascular and inflammatory bowel diseases, and even cancer [84]. Some studies have also associated gut microbiome with intestinal immunity. It has been shown that a healthy microbiota improves local expression of a Toll-like receptor (TLR) [85] which recognizes the PAMPs (pathogen-associated molecular patterns) expressed by a broad range of infective agents and improves the percentage of antigen-presenting cells, differentiated T cells, and lymphoid follicles [86, 87]. Besides the local immunity, the gut microbiota affects the systemic immunity by increasing splenic CD4^+^ T cells and systematic antibody expression [88]. Consequently, the global role of the gut microbiota in intestinal immunity has increased the interest of the scientific community in developing techniques that improve human health by manipulating the gut microbiota.

Nowadays, researchers are exploiting these important results for medical applications; for instance, fecal microbiota transplantation (FMT) has been used to eliminate *Clostridium difficile* recurrent infection by transplantation of healthy microbiota in human patients [89]. Besides that, FMT has been successfully used in treatment of inflammatory bowel disease, functional gastrointestinal disorders, hepatic encephalopathy, obesity, and metabolic syndrome [24, 90].

### 2.6. Biomedical Significance

Findings from studies of the gut microbiome shed a light over a number of diseases directly impacted by it, becoming a promising scope for advances in understanding and treating of complex diseases. Among them, Crohn's disease [91], rheumatoid arthritis [92], obesity [93, 94], type 1 and type 2 diabetes [95–97], breast cancer [98, 99], and atherosclerosis [100] can be cited as associated to the gut microbiome. Thus, researchers are interested in finding biomarkers and microbiome-based signatures for use in diagnostics, prognostics, and treatment of patients with diseases related to the human microbiome, describing important targets with biomedical significance that could be useful for public health.

In this context, Yu and collaborators [101] performed a metagenome-wide association study (MGWAS) using stool samples from 74 Chinese individuals with colorectal cancer and 54 controls, aiming to identify noninvasive biomarkers for colorectal cancer. Authors found that, besides known colorectal cancer-associated species such as *Fusobacterium nucleatum* and *Peptostreptococcus stomatis*, the other 20 microbial gene markers could differentiate colorectal cancer from control patients. In order to define a “worldwide” signature for colorectal cancer identification, they validated four of these gene markers in Danish-, French-, and Austrian-published cohorts. This result indicates that the four biomarkers validated in individuals from different countries might be used to early diagnosis of colorectal cancer even in different populations with different gut microbiome structures and is a promise for early noninvasive diagnosis of the disease. Later, Yu's group [102] developed a new diagnostic tool for colorectal cancer using the four biomarkers validated in 2015. For this, they applied a probe-based duplex qPCR assay for quantifying these bacterial genes and showed that one of these genes can discriminate colorectal cancer from control individuals with 77.7% of sensitivity and 79.5% of specificity. Moreover, combining these four bacterial genes with a fecal immunochemical test improved the diagnostic, displaying a sensitivity of 92.8% and a specificity of 81.5%.

In the same way, Pascal et al. [103] defined a microbial signature for Crohn's disease. They performed a MGWAS using samples from 2045 individuals from four countries (Spain, Belgium, UK, and Germany) and found eight groups of microorganisms that could be used to discriminate between Crohn' disease and ulcerative colitis (the two main inflammatory bowel diseases that share many immunologic, epidemiologic, and clinical features). Then, they developed an algorithm that showed specificity of approximately 90% of Crohn's disease detection when compared with healthy control, anorexia, ulcerative colitis, and inflammatory bowel syndrome patients. Similarly, Loomba et al. 2017 [104] defined a gut microbiome signature for the diagnosis of advanced fibrosis in individuals with nonalcoholic fatty liver disease using a metagenomic analysis. Analogously, a similar approach was used to distinguish between type 2 diabetes individuals and nondiabetic controls [97]. By means of a sequence-based profiling metagenomic approach, authors showed that type 2 diabetes individuals were characterized by an increase of opportunist pathogens, an enrichment of sulphate reduction genes and oxidative stress resistance genes, and a decrease in butyrate-producing species. Taken together, these gut metagenomic markers might become a powerful tool for the diagnosis of patients with the disease.

### 2.7. Mining of Microbial Genes In Vivo

New outcomes from the studies of the human microbiome inspired novel biological questions related to the microbial fitness at diverse human tracts. Thus, elucidating the set of genes involved in the colonization and maintenance of the intestinal microbiota would provide valuable tools to further engineering of probiotics or to enhance the survival of certain microorganisms directly related to healthy conditions.

In this way, the *in vivo* temporal functional metagenomics approach, developed by Yaung and collaborators [105], allowed them to mine microbial genes associated to microbial fitness in the mammalian gastrointestinal tract. The temporal sequencing platform employed in this study allowed the detection of genes that confer microbial fitness using a mouse as a model host. Thus, authors inoculated germ-free mice with *E. coli* transformed with a library composed of 2–5 kb fragments of the *Bacteroides thetaiotaomicron* (Bt) complete genome. Through a 28-day experiment of kinetic monitoring of the enriched clones inside the mice's gastrointestinal tract, they were able to identify genes related to colonization and maintenance of the bacteria inside the tract. The study revealed that different sets of genes were enriched in the pool of bacteria at contrasting times. For instance, during the early phase of colonization, genes responsible for the synthesis of polysaccharides and lipopolysaccharides (LPS) were significantly present in the pool, expanding *E. coli's* LPS biosynthesis repertoire with the acquired Bt biosynthetic genes. Those changes in the LPS synthesis could provide the *E. coli* with different antigenicity and enhance resistance to barriers for colonization in the gut. Nevertheless, a distinct set of genes was present in the long-term experiment, mostly related to sugar metabolism or transport. Furthermore, except for a mutation in the *galK* chromosomal gene, the recipient strain maintained its genetic stability [105].

The work is the first one to use temporal-functional metagenomics to describe how temporal data can contribute to the discovery of genes with functions of interest, once most of the genes involved in the GI tract community fitness would not be found if the data was from only an endpoint [105]. Temporal approaches like these could also bring interesting insights about interaction dynamics and fitness of microbes in other environments (such as bacteria associated to plant growth or parasitic interactions), unraveling the genes involved in a microbial community structure and metabolism. Moreover, identified genes could be used as novel drug target genes in pathogenic bacteria.

### 2.8. Biotechnological Impact

Biotechnology is one of the most favored fields by the metagenomic era. As microorganisms are the major source of biocatalysts for industrial purposes, increasing the repertoire of biochemical transformations available for biotechnological solutions is of high relevance [106]. Since Healy and collaborators [28] introduced the idea of constructing metagenomic libraries from a gene of interest-related environment, functional and sequence-based metagenomics have been shown to be effective in the identification of novel genes that confer resistance to extreme conditions, enzymes, antibiotics, and other bioactive molecules derived from a variety of environments (Table 1) [107–111]. Furthermore, functional metagenomic approaches made it possible to identify novel biological parts with specific activities without the need for isolation and cultivation of microorganisms.

In this context, a report from Ferrer and collaborators [112] should be highlighted. Authors constructed a metagenomic library of DNA from a cow rumen in a phage lambda vector and performed functional screenings for different carbohydrate-active enzymes. Considering that cow rumen microorganisms are specialized in degradation of cellulosic plant material, the sample used for library construction should be enriched in biomass-degrading genes. The success of the approach was exposed by the high rate of recovering of clones with different hydrolytic activities (22 clones), being among them are 9 esterases, 12 endo-*β*-1,4-glucanase, and 1 cyclodextrinase. Moreover, after DNA sequencing analysis of the enzymes, 8 could not be found in any sequenced genomic data, revealing that 36% of the recovered enzymes were completely new.

Other interesting works related to exploring functional genes in leaf-cutter ant fungus gardens were carried out to determine enzymes and pathways involved in symbiosis between leaf-cutter ants and their cultivar. By using metagenomic approaches, authors have determined the microbial composition of the fungus gardens [113, 114] and how the plant biomass degrading process in this microbial community occurs, showing a number of novel cellulases involved in it [113–115].

Alternative relevant metagenomic studies take advantage of the genetic potential of microbe inhabitants of extreme environments, such as high or low temperatures, salinity, acidity, pressure, radiation, or high concentrations of heavy metals (Table 1) [116–118]. Deciphering microbial diversity and metabolic activities of microorganisms in extreme conditions reveals the biochemical strategies used by them to survive under extreme conditions. This, in turn, can be used to expand the capability of survival of bacteria used in industrial processes. In addition, those organisms are interesting sources for enzymatic activities with specific and unusual features. Table 1 summarises some of the most noticeable genes that have been discovered until now using the strategies described above.

## 3. Conclusions and Perspectives

Over the last three decades, since the first studies using the concept of metagenomics, extraordinary advances in the field have been achieved. Collective intelligence from a plethora of experts in diverse fields (such as biologists, biochemists, geneticists, physicists, and computer scientists) was imperative for answering central biological questions and for bringing biotechnological solutions in a myriad of different fields.

Understanding properties such as structure, diversity, richness, and dynamics of microbial communities is essential for unravelling the underlying processes that govern the organization of those systems. However, for a more comprehensive analysis, it is crucial to integrate information from both the microbial community and the environment it is embedded in. The macrodynamics of physical spaces and the interactions between their components directly modulate the microbiological universe (and vice versa). Thus, understanding the physical, chemical, and relational aspects of an environment can provide insightful predictions about its microinhabitants, whereas the reverse process, depicting an environment from a collection of “microbial footprints,” although more challenging, is also attainable [119].

In this context, one of the most proximal models of the study is our own and the built spaces we create and live in. From our bodies to our cities and far away, the Earth is heavily packed with microbes [120–122]. A “reference man” (one who is 70 kilograms, 20–30 years old, and 1.7 meters tall) contains on average about 30 trillion human cells and 39 trillion bacteria [123] and emits bacteria at rates of over a million biological particles per hour [124]. Then, it is daunting to ask can we understand the “maketh men” through its microbiome? Nowadays, we know that our personal ecosystem of microbes is shed on everything we touch and everyplace we go as “molecular echoes.” Thus, can we trace back an individual lifetime through metagenomics? What about the lifetime of a whole society? In reverse, can we use metagenomics to guide the rational design of novel-built environments (indoors and outdoors) for artificially selecting microbial communities, which will ultimately contribute to human health? The answer to all those exciting questions is yes, we can—at least, partially—as it was described in diverse examples along this revision, which are enabling us to move towards the right answers.

In contrast, despite the notable milestones reached in the field, there are still crucial challenges that need to be faced in order to delineate new conceptual advances in microbial science. Development of novel bioinformatic tools specific for metagenomic analysis is still necessary, once the next-generation sequencing platforms are generating an increasing amount of data that is not directly proportional to its biological significance. That is, there is an enormous quantity of information in sequenced data that need to be transformed into biological understating. In the near future, huge data processing and analysis in an integrated way with data already known will be the main challenge of the field [30].

On the other hand, the success of the function-based screenings depends on factors like the size of the gene in metagenomic DNA, its abundance in the samples, the efficiency of the screening method, the host vector system used, and the heterologous expression of the gene. Nowadays, after overcoming of some critical limitations related to host-biased screenings, researchers have used alternative hosts besides *E. coli* to perform the screenings [125–130]. For this, the use of broad host range vectors, able to replicate in different hosts, is required. Although a large collection of broad host range vectors is available [131], we still need to create *a la carte* vectors specific for some microorganisms that are essential to certain industrial uses. In this sense, strategies involving synthetic biological approaches [132] have been crucial to develop new smart screening methods. Engineering biological circuits—the so-called biosensors—have accelerated the identification of positive activities in metagenomic screenings including millions of clones. Interesting examples are the substrate-induced gene expression (SIGEX) and the product-induced gene expression (PIGEX) approaches [17, 133] or a riboswitch-based selection system initially constructed for mining thiamine uptake functions [134], but generalizable to other compounds. Furthermore, developing new methods for expanding the search space of functional metagenomics from enzymes to novel genetic elements such as regulators, promoters, and cis-regulatory sequences is very important for both mining biological parts and understanding their natural diversity [17, 130, 133, 135].

In summary, current challenges in metagenomics that need to be addressed can be divided into two main groups: (i) the development of novel bioinformatic tools and (ii) the generation of novel molecular tools. The first group comprises the necessity of dealing with the colossal amount of information delivered from novel sequencing approaches, as previously described. Thus, it is imperative to transform the metagenomic information overload into biological understanding. The second challenge is related to the generation of novel molecular approaches such as merging metagenomic and synthetic biology to delineate novel strategies for activity-driven screening. Existing functional screening methods usually have low rates of gene target identification. Therefore, the construction of novel synthetic circuits able to detect enzymatic activities—or other target gene output—present in the cloned metagenomic fragments is essential to improve the screening efficiency of metagenomic libraries. In this manner, by combining the collective efforts of specialists for overcoming the previously described challenges, it will be possible to integrate emerging concepts and dive deeper into the universe of metagenomics expanding the current knowledge in a myriad of areas. Shedding a light on the “hidden” world of uncultured microorganisms—and its inherent biochemical treasures—shall tell us stories not only about the multitude of Microverses that surround us but also about ourselves.

## Figures and Tables

**Table 1:** Genes discovered through metagenomic approaches with high biotechnological potential.

Function/gene target	DNA source	Library size	Screening method^∗^	Number of hits found	Biotechnological relevance	Reference
*Enzymes*						
Esterases, endo-*β*-1,4-glucanases, and cyclodextrinase	Cow rumen	1.1 Gb	Function based	22	Eight enzymes (36%) were entirely new	[112]
Laccase	Water from South China Sea	1.4 Gb	Sequencing based	1	High chloride resistance and ability to decolorize industrial dyes	[136]
Naphthalene dioxygenase	Oil-contaminated soil	294 Mb	Function based	2	Applicable in oil-contaminated soil/water	[137]
Oxygenases	Artificially polluted soil	5.2 Gb	Function based	29	Applicable in oil-contaminated soil/water	[126]
Cutinases	Leaf-branch compost	735 Mb	Function based	19	Potential application in polyethylene terephthalate (PET) degradation	[138]
Phenol hydroxylases and catechol 2,3-dioxygenases	Wastewater treatment plant	495 Mb	Function based	413	Potential use in aromatic compound degradation	[139]
Carboxylesterase	Marine water	~1.3 Gb	Function based	95	Cold-active and salt-resistant enzyme	[140]
Cellulase/esterase	Water lakes	1.86 Gb	Function based	3	New cellulase	[141]
Cellulase	Soil	Not found	Function based	1	Halo- and thermotolerant enzyme	[142]
*β*-Glucosidase	Hydrothermal spring water	Not found	Function based	1	Thermotolerant and heath-active enzyme	[143]
Lipase/protease/hemolysins/biosurfactants	Slaughterhouse drain	~884 Mb	Function based	22	Antimicrobial activity	[144]

*Genes that confer resistance to extreme conditions*						
Acid resistance genes	Plankton and rhizosphere from Tinto River	~2.3 Gb	Function based	15	Genes involved in acid resistance	[118]
Nickel resistance genes	Rhizosphere of E. andevalensis from Tinto River	2.15 Gb	Function based	13	Genes related to nickel resistance	[117]
Salt resistance genes	Brine and rhizosphere from Es Trenc saltern	2.15 Gb	function-based	11	Genes conferring salt resistance	[116]
Arsenic resistance genes	Headwater from Tinto River	151 Mb	Function based	18	Genes involved in arsenic resistance	[145]

*Regulatory sequences*						
Constitutive promoters	Soil from secondary Atlantic Forest	~500 Mb	Function based	33	Use as “biobricks”	[135]

*Pathways/systems/operons*						
Naphthalene-degrading system	Naphthalene-contaminated groundwater	~283 Mb	Sequencing based	3	Pollutant-degrading enzyme systems	[146]
Dioxygenase-degrading cluster	Forest soil	260–815 bp	Sequencing based	11	Degrading phenoxyalkanoic acid (PAA) herbicides avoiding groundwater contamination	[147]
NRPS biosynthetic pathway	Tunicate consortium in Florida Keys	~280 Mb	Sequencing based	1	ET-743 biosynthetic pathway; anticancer molecule	[148]

*Bioactive molecules*						
Pigmentation producing and antibacterial activity	Soil	Not found	Function based	45	Potential new molecules to be used as antibiotics	[125]
Turbomycin A and B	Soil	~1 Gb	Function based	3	Antibiotic activity	[16]
Antimicrobial small molecules	Soil	~720 Mb	Function based	4	Antibiotic activity	[149]

^∗^All genes discovered through sequencing-based methodologies were experimentally tested for their related functions.
